# Supplementary material for: The causal relationship between the human gut microbiota and pyogenic arthritis: a Mendelian randomization study
Source: Front Cell Infect Microbiol. 2024 Nov 26;14:1452480. doi: 10.3389/fcimb.2024.1452480 (PMC11629706; doi:10.3389/fcimb.2024.1452480)
Supplement: Supplementary file 1 [file DataSheet1.zip › mendelian test/ebi-a-GCST90027539.csv_forest.pdf]

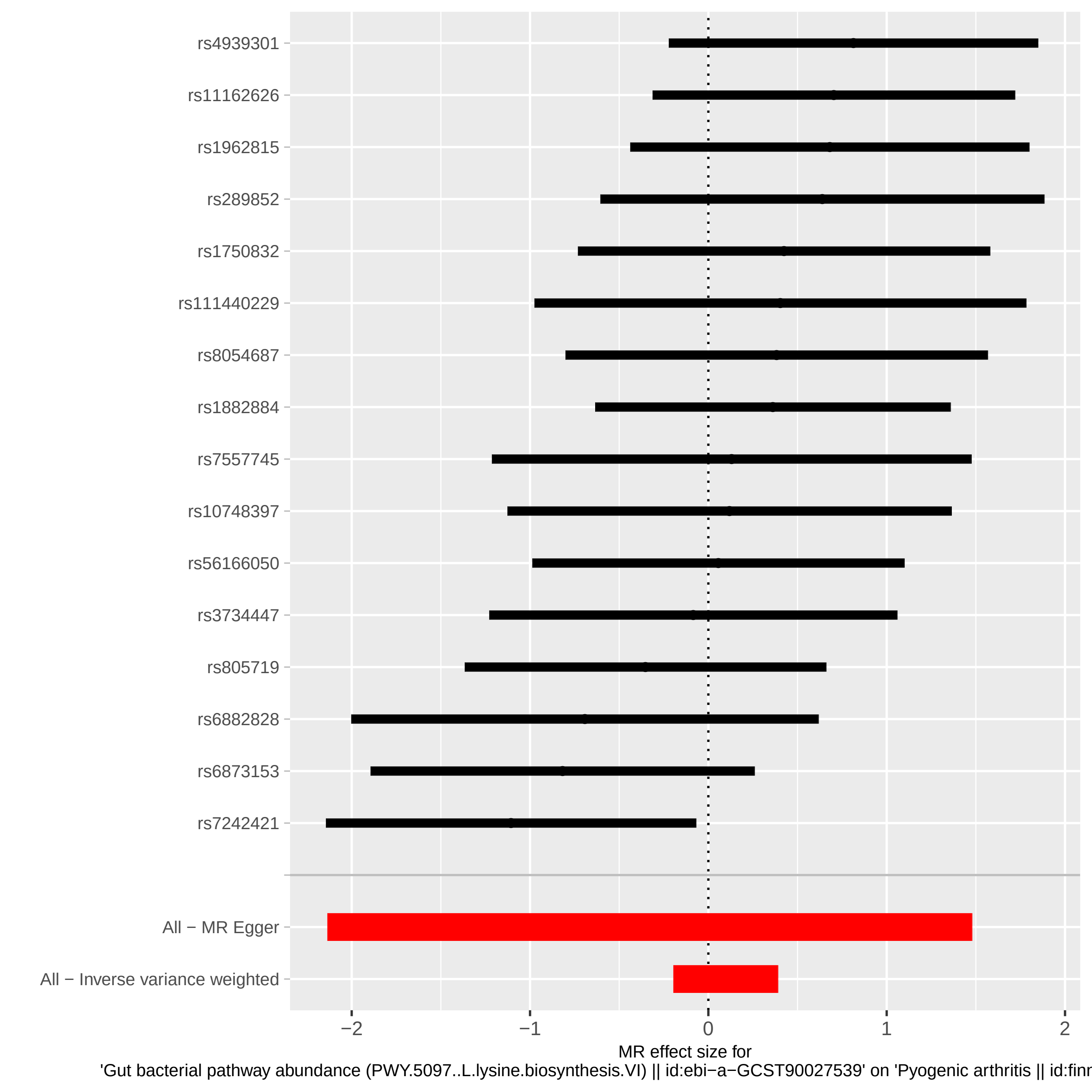

rs4939301
rs11162626
rs1962815
rs289852
rs1750832
rs111440229
rs8054687
rs1882884
rs7557745
rs10748397
rs56166050
rs3734447
rs805719
rs6882828
rs6873153
rs7242421
All − MR Egger
All − Inverse variance weighted
−2
−1
0
1
2
MR effect size for
'Gut bacterial pathway abundance (PWY.5097..L.lysine.biosynthesis.VI) || id:ebi−a−GCST90027539' on 'Pyogenic arthritis || id:finn
